# Wii or Kinect? A Pilot Study of the Exergame Effects on Older Adults’ Physical Fitness and Psychological Perception

**DOI:** 10.3390/ijerph182412939

**Published:** 2021-12-08

**Authors:** Jinhui Li, Long Li, Peng Huo, Cheng Ma, Linlin Wang, Yin Leng Theng

**Affiliations:** 1 School of Journalism and Communication, Jinan University, Guangzhou 510632, China; 2 National Media Experimental Teaching Demonstration Center, Jinan University, Guangzhou 510632, China; 3 Department of Communication, College of Educational Information Technology, South China Normal University, Guangzhou 510631, China; ll851721@163.com; 4 Wee Kim Wee School of Communication and Information, Nanyang Technological University, Singapore 637551, Singapore; phuo001@e.ntu.edu.sg (P.H.); cma001@e.ntu.edu.sg (C.M.); lwang015@ntu.edu.sg (L.W.); tyltheng@ntu.edu.sg (Y.L.T.)

**Keywords:** aging, active video game, physical health, exercise intention, psychological perception

## Abstract

Exergames are now often implemented among older adults for health purposes. This study aimed to investigate whether playing Kinect and Wii exergames has effects on older adults’ physical fitness and psychological perceptions towards exergames. A total of 23 older participants aged above 60 years were recruited and randomly assigned into two groups, in which they played either Kinect or Wii Bowling exergames for three sessions in one week. Physiological and psychological measures were collected including heart rate, blood pressure, shoulder flexibility, as well as perceived benefits and intentions for future use. Findings indicated that exergames are equivalent to light-intensity exercises, and hence pose no or minimal risk to older adults. Older adults had a positive attitude towards exergames and have a strong willingness to engage in exergaming on a regular basis. Although no significant platform difference was identified, observation and qualitative findings suggested that Wii might provide a more intense physical activity than Kinect, while Kinect might obtain a higher perception among older adults than Wii. The study has several practical implications for both health professionals and exergame designers targeting the ageing population.

## 1. Introduction

The world population is rapidly ageing. The issue of an ageing population characterized by both longer life expectancy and declining fertility rate has been a growing concern among researchers and policymakers [1]. With people living ever longer, the challenge is to focus on research that allows the elderly not just to survive, but also to stay healthy [2]. Studies indicate that social isolation and lack of regular exercise are amongst the prime factors for deteriorating health status and potential health risk for the elderly [3]. They show that the elderly with a low level of social participation tend to stay at home and have a higher propensity to depression symptoms, which could impair their physical fitness and require clinical attention. The inability to exercise regularly reduces their physical fitness and imposes a sedentary lifestyle which leads to serious pathophysiological consequences, including muscle atrophy, impaired balance, orthostatic hypotension, and impaired cardiorespiratory function, as well as to psychological consequences such as apathy, depression and cognitive decline [4].

Over the years, the gaming industry has evolved quickly with the advancements in interactive digital technologies, and digital games no longer just target teenagers or game enthusiasts, but are designed for a wider range of age groups, including the elderly population [5]. The concept of exergames was introduced in the 1980s, which represented the trend of using consumer digital games for actual exercise [6]. Players are allowed to play games using body gestures or motion-sensing devices and gain sufficient workout while playing games with each other. It had been suggested that exergames might serve to counteract sedentary lifestyles and enhance individual health behavior [7]. The advent of motion-detection technologies in exergaming has helped to expand the beneficiary group of exergaming to the elderly [8]. Light to moderate intensity of exercise is often recommended for older adults to maintain good health status and provide sufficient workout for their body [9]. However, older adults often do not have adequate access to exercise opportunities and refrain themselves from outdoor exercises [10]. As such, exergaming appears to be a suitable substitute for traditional forms of exercise as it can be conveniently done at home. In recent practice, exergaming has often been implemented as various kinds of health interventions in both home and community settings, to improve physiological well-being [11,12]. A systematic review from Peng et al. [13] evaluated these existing studies and found a positive effect of exergames on promoting physical activity. Furthermore, an increasing number of studies have been undertaken to measure the health effects for the group of older adults, including physical, cognitive, mental, and social benefits [14,15,16].

Among the various exergaming platforms, Nintendo Wii (or ‘Wii’) and Microsoft Xbox 360 Kinect (or ‘Kinect’) are the two mainstream ones in the current market. Both have been designed essentially as interactive games between computer and player as well as among the players themselves. Many previous studies have implemented or tested the impact of exergames based on these two platforms [17,18,19]. Despite the similarity, there are important differences between Wii and Kinect in both connection mechanism and form of play. Wii was first released in 2006 and utilized a motion sensitive controller to detect three-dimensions (3D), and the 2010 version of the Wii could also detect a player’s 3D hand posture using a three-axis gyro sensor. On the other hand, Kinect is a totally controller-free console that supports motion control and voice control by infrared projector and camera [20]. The lack of a handheld controller by Kinect provides for greater freedom of movement and thus higher levels of physical activity than Wii. Findings from O’Donovan et al. [21] have supported this claim and indicated that playing on Kinect elicited greater energy expenditure than playing on Wii among young healthy adults. Yet no significant differences between Kinect and Wii were found on heart rate, oxygen consumption, and ventilation among college students [22]. The technological differences across the two platforms may further result in different perception and preferences among users. Since exergaming is still a relatively new concept, the potential impacts have remained unknown of different exergaming platforms, such as Wii and Kinect, among older adults.

This paper, therefore, mainly attempts to establish a relationship between exergame platforms and physical fitness among older adults. Specifically, it investigates whether Wii and Kinect exergames would have different impacts on physiological fitness, perception, and usage intention among older adults.

## 2. Materials and Methods

A between-group experiment was conducted to assess the impact of playing Wii and Kinect games. Both quantitative and qualitative measurements were involved to monitor the change in physiological parameters such as heart rate and blood pressure and collect psychological parameters such as the participants’ perception and intention towards playing exergames. The study obtained ethical approval from Nanyang Technological University (IRB11/06/08) where the study was originally conducted.

### 2.1. Participants

The primary inclusive criteria of the recruited participants in this study was aged 60 and above, as specified by the World Health Organization [23] for an elderly group. Meanwhile, health screening was conducted before the actual study to assess the eligibility of included participants. They had to have a systolic blood pressure <130–159 mm Hg and a diastolic blood pressure <95 mm Hg (not on antihypertensive medication). Participants were excluded if they had cognitive disability or mobility limitations to play Wii or Kinect games. They were recruited through the help of a local senior activity center in Singapore. G*Power was used for the sample size calculation. Based on a priori power analysis, a sample of 24 participants is required to determine a large effect size of f = 0.6 with a power of 0.8 (α = 0.05). Therefore, a total of 23 (16 females and 7 males) participants volunteered to take part in our study, who are active members of the center. The sample size was consistent with other existing exergaming studies [24,25]. The age of the participants ranged from 64 to 84 years old, and all of them were in good health condition and capable of some light to moderate intensity of exercise. The participants were asked to sign the consent form and were given a briefing prior to the commencement of the study. As a token of appreciation, each participant who completed the whole study was awarded SGD$10 equivalent of shopping voucher.

### 2.2. Intervention

For the game selection, we considered the safety and energy expenditure for older adults. The game chosen also needed to be compatible on both Wii and Kinect platforms. Referring to the existing work, bowling, golf, and tennis are often involved in various elderly studies related to game playing. Particularly bowling has been used to evaluate the effects of digital games for frail elderly persons [26], as well as for the study of elderly person’s experience and preference on motion sensing controllers [27]. The bowling game requires minimum lower limb movement, also referred to as a template when programmers design new exergames for elderly people. In the theoretical research of exergaming for elderly users, bowling is also recommended, as it is self-paced and intuitive to play [5]. More importantly, the bowling games are available on both the Kinect and Wii platforms. As a result, we selected bowling games in *Wii Sports* and *Kinect Sports* as the interventions in the current study.

### 2.3. Allocation and Procedure

**Figure 1:**
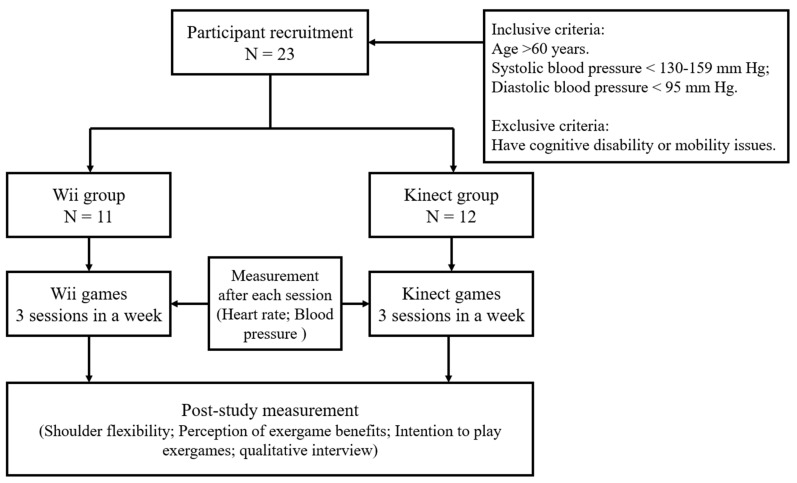
Study flowchart.

To prevent selection bias, participants were randomly assigned into two experimental conditions (Wii or Kinect) at the beginning of the experiment. Considering the relatively small sample size, we mainly applied simple randomization with gender stratification, in order to have an equal mix of males and females in each condition. The allocation process also enabled blinding which kept trial participants and game facilitators unaware of the assigned intervention, so that they would not be influenced by that knowledge. In the final study, a total of 23 participants was randomly divided into Wii group (*n* = 11) and Kinect group (*n* = 12). One facilitator was assigned in each group to calibrate the console as well as take the various measurements for the participants. Each participant was asked to attend three sessions in a week. All the sessions were conducted in the local senior activity center. In every session, participants were led into the playroom by one of the facilitators according to the arranged sequence and then played the game for about 15 min. This duration is a baseline required for the elderly to achieve light to medium intensity of exercise after which we then recorded the effects of the physiological workload incurred during the exercise with selected fitness indicators. After the elderly participants had completed their sessions, the facilitator measured again their blood pressure and heart rate. The entire session for each participant lasted about half an hour. The flowchart of this study is shown in Figure 1.

**Figure 2:**
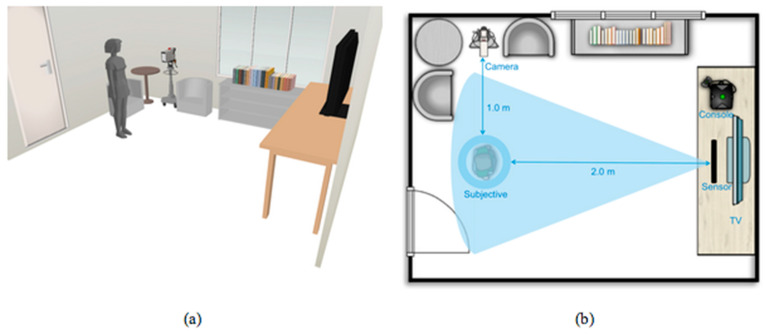
Room settings of the study environment. Note: (**a**) Third person view; (**b**) Top view.

Both the Kinect and Wii participant groups had their sessions held concurrently in separate venues. Two video cameras with a tripod stand to record the entire game session for each participant were set up, and the cameras had been calibrated to capture the shoulder range of motion. In addition, two sofa seats were arranged in the rooms to provide the participants with a comfortable setting to take their heartbeat and blood pressure measurements. The room setup is illustrated in Figure 2.

### 2.4. Measurements

*Heart Rate (HR)*. HR is the number of heart beats per minute, and the accepted norm is age-related. While exercising, the highest HR that a healthy individual can achieve is defined as Maximum HR (HRmax), which can be theoretically estimated by HRmax = 220 minus age [28]. For example, if a participant is 70 years old, his/her HRmax = 220 − 70 = 150. Measuring the exercise HR as the percent of HRmax (%HRmax) is the easiest and most effective method to indicate exercise intensity, and exercise intensity is the one of the most important factors for maintaining and developing cardiorespiratory fitness [29]. The exercise intensity has been categorized by Panton et al. [30] as follow: (i) Vigorous intensity is 80–90% of HRmax; (ii) Moderate intensity is 60–79% of HRmax; and (iii) Light intensity is 35–59% of HRmax. Moderate intensity is usually considered as the target HR zone for this participant’s age group as they gain the most benefit and minimal risks when they exercise within their target HR zone. Hence, by monitoring HR, we can determine whether playing Wii or Kinect can satisfy the workout needs for elderly participants. HR was measured by a digital wrist sphygmomanometer in the current study.

*Blood Pressure (BP).* BP was to monitor the status of the participants and the experiment were controlled so that elderly participants did not exercise beyond their limit. Analyzing BP was able to determine if exergaming posed any danger to the elderly participants, such as an abnormal surge in BP that exceeded the healthy range. In addition, it served as an extra precaution to ensure that the elderly participants would go through the experiments without suffering from any physical or mental damage. BP included Systolic Blood Pressure (SBP) and Diastolic Blood Pressure (DBP), which were also measured by a digital wrist sphygmomanometer in the current study.

*Shoulder Flexibility (SF)*. SF measures the degree between the angles that the arm swings around the body. This is a relatively new research area on the possible benefits that can be derived from exergaming. It is measured from the highest point that the arm reaches at the back of the body to the highest point the arm can be lifted to in the front. SF helps find out if older adults who played Wii or Kinect exergames are capable of stretching to a larger angle after a few sessions. It is also an indicator for physical improvement. For the SF measurement, we mainly applied the computer software “Screen Protractor” to calculate the range of motion from the screenshots randomly captured by the video camera throughout the experiment, and results were assessed combining all the sessions. This measurement was adapted from previous studies [31,32] where the validity and reliability had been examined.

*Perception of Exergame Benefits (PEB)*. This assesses participants’ perception on the benefits of exergames to their health according to the President’s Council on Physical Fitness and Sports definition [33]. Three perceptual aspects relevant to the study were chosen from the definition, including cardiovascular fitness, flexibility, and muscle strength. They were measured by a post-exergaming survey with a five-point Likert from “Strongly Disagree (1)” to “Strongly Agree (5)”.

*Intention to Play Exergames (IPE).* This was designed to investigate participants’ degree of willingness to play Kinect or Wii exergames, and the elderly’s routine for physical exercise. This measurement was adapted from the Physical Activity Questionnaire for Elderly Japanese [34]. The responders fill in their intended frequency and duration of engagement in physical activities or exergames. For the frequency, there are four categories on a weekly basis: never, seldom (1 or 2 days), sometimes (3 or 4 days), or often (5–7 days) and the options of the duration are separated to <30 min, 30 min to <1 h, 1 to 2 h, and more. The answers to the different categories are converted to scores and computed. The score of willingness to do exercise was calculated by multiplying the frequency and duration. Before the intervention, the responders were asked to rate the frequency and duration of current physical activity and after intervention the responders rated the frequency and duration of their willingness to engage Wii or Kinect.

The cardiovascular responses of the participants including HR and BP were measured before and after each session to provide empirical evidence of any physiological impact from exergaming. SF were collected during the three sessions. All the results obtained from the first session were used as a control set to be compared with the results obtained from the second and third sessions. Each participant had his or her performance monitored and recorded to facilitate assessment on an individual basis. The psychological variables of PEB and IPE were collected though a post-study survey with paper questionnaire. Meanwhile, a one-to-one interview was further conducted on all the participants after they completed the intervention, to provide a deeper understanding of their perception and intention towards playing exergames on Wii or Kinect.

### 2.5. Data Analysis

All the data were analyzed through IBM SPSS version 25. Descriptive statistics was first performed, followed by the assumption checks for normality and homogeneity. The results from the Shapiro–Wilk test indicated that all key variables of HR, SBP, DBP, SF, PEB, and IPE appeared to be normally distributed (*p* > 0.05) in the two experimental groups. The results from Levene’s test further demonstrated that these variables had the same variance *(p* > 0.05) across the two experimental groups. A series of Pearson Chi-square tests and Mixed Analysis of Variance (ANOVA) with post hoc multiple comparisons by Bonferroni correction was used to compare the difference in the two groups. Bonferroni correction shows a detailed relation between each of the two groups, with minimum Type I error brought by α (where = (*n*, 2)).

## 3. Results

### 3.1. Physiological Well-Being

The mean age of the 23 participants in the study was 72.47 (SD = 5.93). From the findings, 94.44% of the Kinect participants and 84.85% of the Wii participants reached the HR equivalent of light to moderate intensity. The findings supported that both Kinect and Wii exergames were able to provide light to moderate exercise intensity for older adults in a relatively short time of 15 min. Surprisingly, a higher percentage of Wii participants reached moderate intensity compared to that of Kinect participants, while a lower percentage of Wii participant reached light intensity compared to that of Kinect participants. Nevertheless, the result from the Pearson Chi-square test did not support the statistical significance of observation, with χ^2^ (1) = 1.84, *p* = 0.20. Table 1 indicates the number of participants of different exercise intensity across the three sessions.

SBP and DBP were assessed to find out whether exergaming with Kinect and Wii would raise participants’ blood pressure to an unhealthy range. The American Heart Association has defined that systolic measurements normally max out around 190 to 220 Hg mm and diastolic blood pressure should remain near or just under the resting measurement during exercise. Results from the three sessions showed that the average SBP at post-test period were 134.31 (SD = 15.19) Hg mm for the Kinect group and 133.85 (SD = 18.23) Hg mm for the Wii group, respectively. Average DBP at post-test period were 88.47 (SD = 11.45) Hg mm for the Kinect group and 87.97 (SD = 10.58) Hg mm for the Wii group, respectively. Mixed ANOVA tests revealed exergame playing (combining Kinect and Wii groups) led to a significant difference between pre-test and post-test DBP (F (1, 21) = 11.053, *p* = 0.003, partial *η* = 0.345), as well as DBP (F (1, 21) = 9.899, *p* = 0.005, partial *η* = 0.320). However, no significant interaction effect was found between time (pre-test vs. post-test) and platform (Kinect vs. Wii) among DBP (F (1, 21) = 0.162, *p* = 0.692, partial *η* = 0.008) and SBP (F (1, 21) = 0.645, *p* = 0.431, partial *η* = 0.030). One-way ANOVA further suggested that the Kinect group did not have a significant difference between pre-test and post-test DBP (F (1, 11) = 3.26, *p* = 0.098), while the Wii group had a significant difference between pre-test and post-test DBP (F (1, 10) = 11.19, *p* = 0.007). Table 2 shows the BP measurement during the three sessions. Furthermore, only one case from the Wii group (around 3% of the total cases) had SBP that reached around 195 Hg mm whereas no one from the Kinect group reached the hypertension stage. Taken together, although exergames are unlikely to cause hypertension or any potential cardiovascular risk on older adults, Kinect exergames may have an even smaller chance when compared to Wii exergames.

**Figure 3:**
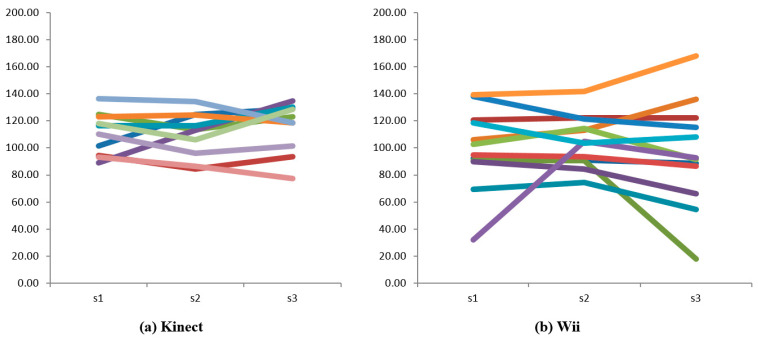
Individual trend of shoulder flexibility. Note: Each color line refers to an individual participant.

The average angle of shoulder flexibility was 112.08 degrees (SD = 14.43) for the Kinect group and 99.94 degrees for the Wii group, yet there was no significant difference across the two groups, F (1,20) = 1.74, *p* = 0.202. Furthermore, mixed ANOVA did not support a significant difference of shoulder flexibility across the two groups over the three sessions, with a non-significant interaction effect of F (2, 32) = 1.082, *p* = 0.338, partial *η* = 0.051. Despite the non-significant change over time, 10 participants (5 from Kinect and 5 from Wii group) were observed to have an increase in their average shoulder range of motion in session 2 compared to session 1, and eight participants (3 from Kinect and 5 from Wii group) had an increase in session 3 compared to session 2. Findings from observation also showed that the range of shoulder flexibility for the Wii group was more distributed compared to that for the Kinect group (see Figure 3).

### 3.2. Psychological Perception

From the one-way ANOVA tests, the result shows that both Kinect and Wii have a significant impact on the elderly’s perception of health improvement, making them think they are getting more flexibility in shoulder motion (Kinect: *p* < 0.001; Wii: *p* < 0.001) and stronger muscle power (Kinect: *p* = 0.003; Wii: *p* < 0.001) after playing the exergame, when compared to a “neutral” group with default value of “3” for each question. No significant differences were identified among the perceived benefits between Kinect and Wii exergames on improving cardiovascular fitness *(p* = 0.416), flexibility *(p* = 0.235), and muscle strength *(p* = 0.862). Although quantitative findings did not indicate the difference between the two exergame platforms, qualitative comments from participants showed that they considered Kinect less helpful for cardiovascular purposes as it required less effort to play. They did not consider Kinect as aerobically consuming as Wii games. Comments from two Kinect participants sum up the experiences:

*“I feel easy to throw the ball without holding the controller and the ball can directly go in the direction in which I point.” [Kinect Participant 3, 73 years old]*

*“I do not feel tired after playing 3 rounds, and I can even play another 3 rounds.” [Kinect Participant 7, 72 years old]*

For the intention of exergame usage, participants in the Kinect group had indicated a higher level of willingness to play exergames (Mean = 5.91, SD = 5.02) than doing traditional physical exercise (Mean = 5.01, SD = 4.17). Similarly, participants in the Wii group had the same tendency of showing a higher willingness to play exergames (Mean = 3.54, SD = 1.58) than doing traditional physical exercise (Mean = 3.09, SD = 1.62). Nevertheless, Paired *T*-test findings did not support the significant difference in their preference, with *p* = 0.625 in the Kinect group and *p* = 0.435 in the Wii group. As a result, we cannot prove that the elderly persons were willing to exercise more with exergames compared to physical exercises. Qualitative findings indicated that older adults perceived the same importance for both exergaming and physical exercise, as commented by some participants:

*“I both like Wii and walking, because playing Wii allows me to do exercise at home and walking is the other exercise for outside. If raining I can stay at home to do exercise or else I can go outside to do exercise in the fresh air.” [Wii Participant 5, 70-year old]*

*“It is convenient to do exercise indoors but sometimes going outside to get fresh air that is important too.” [Kinect Participant 5, 69-year old]*

*“I am willing to do exercise with Wii but also need to keep walking exercise.” [Wii Participant 7, 79-year old]*

In addition, there was no statistical significance in the preference between Kinect and Wii though the Mann–Whitney U-Test, with *p* = 0.428. However, results from the qualitative interviews did show the possible impact of technological differences on older adults’ intention to use exergames. Participants from the Kinect group displayed a great willingness to play exergames, while those in the Wii group were frustrated by the control of Wii:

*“I am happy to play Kinect as it gives me more confidence when I make a strike!” [Kinect Participant 8, 84-year old]*

*“I always forget to release the button after the swing (arm).” [Wii Participant 9, 64 year-old]*

*“I always release the button too early before swing (my arm).” [Wii Participant 10, 80-year old]*

## 4. Discussion

Older adults commonly lack physical fitness and hence suffer from severe mobility problems in their upper and lower extremities [10]. The direct benefit of this study was to establish the positive effects of exergaming on heart rate, blood pressure, and shoulder flexibility. The study also examined whether Kinect and Wii bowling games have different influences on older adults’ physical fitness and psychological perception towards exergames. The cardiovascular response to playing the Wii and Kinect bowling game was first examined. In the study, the participants consistently reached light to moderate intensity HR values while playing exergames, equivalent to the recommended physical exercise amount for the elderly to maintain a balanced and healthy lifestyle. Furthermore, most of them approached an HR level where, theoretically, an increase in cardiovascular fitness could be achieved with continuous play [35], and was equivalent to achieving light exercise intensity. The findings were consistent with previous studies which indicated that exergaming has elicited light-to-moderate intensity activity among other populations, such as overweight individuals [36], adolescents [37], and college students [38]. Thus, the cardiovascular benefits of exergames were further extended to older adults who often have a high risk of cardiovascular illness. In terms of blood pressure, none of the elderly participants had a sudden surge in blood pressure and their blood pressures remained within the safe range. Although it is not possible to assume that exergaming is totally risk-free for older adults, the study suggested the risk to be rather minimal as feedback from the elderly participants did not indicate any discomfort during the study. A review from Klompstra et al. [39] also supported the safety of exergame platforms on older adults, by reporting no adverse events occurring in the included studies.

Due to the absence of empirically derived estimates of minimum clinically important differences, Hsu et al. [40] made an a priori decision that a 10-degree improvement in shoulder forward flexion would represent a meaningful change. Although continuous whole-body movement would be expected to result in high energy expenditure and heart rate, the elderly participants did not show significant improvement in their shoulder range of motion. Our recorded results show fluctuations and inconsistency in the exhibited shoulder range of motion even for the same participants. There are several potential explanations for this. First, the non-significant results might be due to the short experiment phase, while we cannot rule out the possibility that exergames could have a positive impact on elderly shoulder flexibility in the long run. As from the qualitative report, the majority of older adults were aware of their arms being stretched which shows that exergaming might indeed improve their shoulder range of motion in the long run and warrants further research. Second, the participants tended to be more casual when engaged with exergames as it is designed for them to relax. Meanwhile, exergames are not so strict as to how the elderly play the games and once they grasped the way the game was played, they could play with much less effort.

Older adults were also reported to have a positive attitude towards the physical benefits of exergames and expressed high willingness to use exergame exercise in the future. Despite exergames being initially designed for the young generation, many studies have found that the ageing population is also interested in playing exergames [41]. A study in Singapore also suggested a high acceptance of exergames among older adults [42]. The evidence has supported exergames as being a suitable intervention for motivating seniors to exercise more. However, the results also show that they still valued traditional ways of exercise that cannot be replaced with exergames. There are other important elements in traditional exercise, such as the embrace of nature or even social interaction with family and friends. Thus, how to incorporate these elements in the future exergame design will need to be addressed.

Regarding the platform differences, no significant impacts were identified between Kinect and Wii exergames on older adults’ physical fitness and psychological perceptions. In spite of different technological designs, both Kinect and Wii platforms achieved similar physiological and psychological outcomes. Consequently, the exergame platform is not supported as being an important factor that influences the benefits for older adults and their perceptions towards exergames. Although not reaching statistically significance, observation and qualitative findings suggested that Wii might provide a more intense physical activity than Kinect, while Kinect might obtain a higher perception among older adults than Wii. The results recommend that older adults with different exercise capacity should use different exergaming consoles. For those who are capable of higher exercise intensity, Wii is recommended to be used, as it provides a more intense game experience and requires better hand-eye coordination. For the elderly who have decreased motor skills or less endurance for physical exercise, they would be advised to use Kinect, because of its easy and intuitive operation.

There were, however, some limitations that could have affected the findings. First, the most difficult challenge of the study is the small number of participants with unbalanced gender condition. The findings on the platform comparison were less valuable when generalized into other contexts. Second, the age range of the study might be too wide given a small group of participants—the physical and mental conditions between elderly aged 64 and 84 years can be extremely different. A larger sample of older adults with balanced demographic and social backgrounds are proposed in a future study with random grouping. Third, we used a between-group study design so that participants did not get a chance to play on both consoles, and this could affect the platform comparison, particularly on the subjective perceptions. Third, the study only involved one type of exergame to ensure equal comparison between the two platforms. It is advisable for future studies to consider more diverse games other than bowling, in order to enhance the generalizability of the findings. Lastly, the key conclusions need to be interpreted with caution given the constraints of time and resources in the current study. A continuous long term tracking study would be preferred to explore the pattern and trend induced by Kinect or Wii, particularly the fitness indicators.

The study has several practical implications for health researchers targeting the improvement of quality of life among the ageing population. First, older adults are advised to engage in exergaming for at least 15 min for each session and three times a week to explore the benefits from exergaming, which provide sufficient physical exercise for the elderly as recommended by ACSM [43]. Second, although exergames would not replace physical exercise, they can still serve as a good substitute for indoor exercise and provide older adults with a safer form of practice compared to outdoor exercise. Third, we would recommend exergame designers to impose certain levels of difficulty and challenges to the games developed so that the elderly exert more energy and are motivated to carry on playing. The measurements used in this study also contribute to a practical guideline to evaluate the influence of new exergames on older adults’ health conditions. Finally, this study also represents an interesting starting point to investigate the potential different impacts of various exergaming platforms, which could inspire further research on the newly platforms such as Nintendo Switch or PlayStation VR.

## Figures and Tables

**Table 1:** Number of participants of different exercise intensity.

Exercise Intensity (by %HRmax)	Session 1		Session 2		Session 3		Average Percentage	
	Kinect	Wii	Kinect	Wii	Kinect	Wii	Kinect	Wii
Vigorous and above (>80%)	1	1	1	2	0	1	5.56%	12.12%
Moderate (60–79%)	1	3	1	2	2	2	11.11%	21.21%
Light (35–59%)	10	7	10	6	10	8	83.33%	63.64%
Less than light (<34%)	0	0	0	1	0	0	0%	3.03%
Total	12	11	12	11	12	11	100%	100%

**Table 2:** Blood pressure measurement across the three sessions.

Time	Session 1		Session 2		Session 3		Average		Group Comparison
	Kinect	Wii	Kinect	Wii	Kinect	Wii	Kinect	Wii	
SBP Pre-test	135.25 (20.44)	121.55 (20.34)	130.42 (15.87)	128.73 (14.28)	121.50 (18.44)	124.73 (19.79)	129.06 (16.00)	125.00 (16.48)	*p* = 0.556
SBP Post-test	142.92 (21.72)	137.36 (23.52)	132.00 (14.47)	134.64 (22.02)	128.00 (21.54)	129.55 (15.83)	134.31 (15.19)	133.85 (18.23)	*p* = 0.948
DBP Pre-test	88.75 (14.28)	80.55 (11.24)	85.50 (10.89)	84.45 (10.25)	79.33 (13.49)	83.82 (11.77)	84.53 (11.36)	82.94 (9.72)	*p* = 0.723
DBP Post-test	95.25 (17.44)	89.55 (15.11)	86.58 (10.02)	88.09 (14.76)	83.58 (15.13)	86.27 (10.09)	88.47 (11.45)	87.97 (10.58)	*p* = 0.914

Note. Data was presented as Mean (SD).

## References

[1] Lee R., Mason A., Lee R., Mason A., Amporfu E., An C.-B., Bixby L.R., Bravo J., Bucheli M., Chen Q. (2014). Is low fertility really a problem? Population aging, dependency, and consumption. Science.

[2] Butler R.N. (1997). Population aging and health. BMJ.

[3] Chan A., Ostbye T., Malhotra R., Malhotra C. Social Isolation, Health and Lifestyles Survey 2009. http://hssr.duke-nus.edu.sg/projects/social-isolation-health-and-lifestyles-survey-2009.

[4] Blocker W. (1992). Maintaining functional independence by mobilizing the aged. Geriatrics.

[5] Ijsselsteijn W., Nap H.H., Kort Y.D., Poels K. Digital game design for elderly users. Proceedings of the 2007 Conference on Future Play.

[6] Sinclair J., Hingston P., Masek M. Considerations for the design of exergames. Proceedings of the 5th International Conference on Computer Graphics and Interactive Techniques in Australia and Southeast Asia.

[7] Rohr L.E., Card A. Impact of active schools interventions in St. John’s, NL. Proceedings of the International Society for Behavioural Nutrition and Physical Activity Conference.

[8] Larsen L.H., Schou L., Lund H.H., Langberg H. (2013). The Physical Effect of Exergames in Healthy Elderly—A Systematic Review. Games Health J..

[9] Mazzeo R.S. (1998). ACSM position stand on exercise and physical activity for older adults. Med. Sci. Sports Exerc..

[10] McPhee J.S., French D.P., Jackson D., Nazroo J., Pendleton N., Degens H. (2016). Physical activity in older age: Perspectives for healthy ageing and frailty. Biogerontology.

[11] Toulotte C., Toursel C., Olivier N. (2012). Wii Fit® training vs. Adapted Physical Activities: Which one is the most appropriate to improve the balance of independent senior subjects? A randomized controlled study. Clin. Rehabil..

[12] O’Connor T.J., Fitzgerald S.G., Cooper R.A., Thorman T.A., Boninger M.L. (2001). Does computer game play aid in motivation of exercise and increase metabolic activity during wheelchair ergometry?. Med. Eng. Phys..

[13] Peng W., Crouse J.C., Lin J.H. (2013). Using active video games for physical activity promotion: A systematic review of the current state of research. Health Educ. Behav..

[14] Stojan R., Voelcker-Rehage C. (2019). A Systematic Review on the Cognitive Benefits and Neurophysiological Correlates of Exergaming in Healthy Older Adults. J. Clin. Med..

[15] Li J., Erdt M., Chen L., Cao Y., Lee S.-Q., Theng Y.-L. (2018). The Social Effects of Exergames on Older Adults: Systematic Review and Metric Analysis. J. Med. Internet Res..

[16] Pacheco T.B.F., de Medeiros C.S.P., de Oliveira V.H.B., Vieira E.R., de Cavalcanti F.A.C. (2020). Effectiveness of exergames for improving mobility and balance in older adults: A systematic review and meta-analysis. Syst. Rev..

[17] Vernadakis N., Papastergiou M., Zetou E., Antoniou P. (2015). The impact of an exergame-based intervention on children’s fundamental motor skills. Comput. Educ..

[18] Bell C.S., Fain E., Daub J., Warren S.H., Howell S.H., Southard K.S., Sellers C., Shadoin H. (2011). Effects of Nintendo Wii on Quality of Life, Social Relationships, and Confidence to Prevent Falls. Phys. Occup. Ther. Geriatr..

[19] Brichetto G., Spallarossa P., Carvalho M.L.L., Battaglia M.A. (2013). The effect of Nintendo® Wii® on balance in people with multiple sclerosis: A pilot randomized control study. Mult. Scler. J..

[20] Microsoft Kinect Setup. http://support.xbox.com/en-US/xbox360/kinect/kinect-sensor-setup.

[21] O’Donovan C., Hirsch E., Holohan E., McBride I., McManus R., Hussey J. (2012). Energy expended playing Xbox Kinect^TM^ and Wii^TM^ games: A preliminary study comparing single and multiplayer modes. Physiotherapy.

[22] Scheer K.S., Siebrant S.M., Brown G.A., Shaw B.S., Shaw I. (2014). Wii, Kinect, and Move. Heart Rate, Oxygen Consumption, Energy Expenditure, and Ventilation due to Different Physically Active Video Game Systems in College Students. Int. J. Exerc. Sci..

[23] WHO Definition of an Older or Elderly Person. http://www.who.int/healthinfo/survey/ageingdefnolder/en/index.html.

[24] Dove E., Astell A.J. (2019). Kinect Project: People with dementia or mild cognitive impairment learning to play group motion-based games. Alzheimer’s Dement. Transl. Res. Clin. Interv..

[25] Yang C.-M., Chen Hsieh J., Chen Y.-C., Yang S.-Y., Lin H.-C.K. (2020). Effects of Kinect exergames on balance training among community older adults: A randomized controlled trial. Medicine.

[26] Gerling K.M., Schulte F.P., Masuch M. Designing and evaluating digital games for frail elderly persons. Proceedings of the 8th International Conference on Advances in Computer Entertainment Technology.

[27] Pham T.P., Theng Y.L. Game controllers for older adults: Experimental study on gameplay experiences and preferences. Proceedings of the International Conference on the Foundations of Digital Games.

[28] Fox S.M. (1968). Physical activity and the prevention of coronary heart disease. Bull. N. Y. Acad. Med..

[29] Hickson R.C., Foster C., Pollock M.L., Galassi T.M., Rich S. (1985). Reduced training intensities and loss of aerobic power, endurance, and cardiac growth. J. Appl. Physiol..

[30] Panton L.B., Graves J.E., Pollock M.L., Garzarella L., Carroll J., Leggett S., Lowenthal D., Guillen G. (1992). Relative heart rate, heart rate reserve and oxygen uptake during exercise in the elderly. J. Gerontol. Ser. A Biol. Sci. Med Sci..

[31] Sahu D., Shah D., Joshi M., Shaikh S., Gaikwad P., Shyam A. (2021). Validation of an on-screen application-based measurement of shoulder range of motion over telehealth medium. J. Shoulder Elbow Surg..

[32] Werner B.C., Holzgrefe R.E., Griffin J.W., Lyons M.L., Cosgrove C.T., Hart J.M., Brockmeier S.F. (2014). Validation of an innovative method of shoulder range-of-motion measurement using a smartphone clinometer application. J. Shoulder Elbow Surg..

[33] Corbin C.B., Pangrazi R.P. Definitions: Health, Fitness, and Physical Activity. The President’s Council on Physical Fitness and Sports Research Digest. https://eric.ed.gov/?id=ED470696.

[34] Yasunaga A., Park H., Watanabe E., Togo F., Park S., Shephard R.J., Aoyagi Y. (2007). Development and evaluation of the physical activity questionnaire for elderly Japanese: The nakanojo study. J. Aging Phys. Act..

[35] Garber C.E., Blissmer B., Deschenes M.R., Franklin B.A., Lamonte M.J., Lee I.M., Nieman D.C., Swain D.P. (2011). Quantity and quality of exercise for developing and maintaining cardiorespiratory, musculoskeletal, and neuromotor fitness in apparently healthy adults: Guidance for prescribing exercise. Med. Sci. Sports Exerc..

[36] Höchsmann C., Schüpbach M., Schmidt-Trucksäss A. (2016). Effects of Exergaming on Physical Activity in Overweight Individuals. Sports Med..

[37] Graves L., Stratton G., Ridgers N.D., Cable N.T. (2008). Energy expenditure in adolescents playing new generation computer games. Br. J. Sports Med..

[38] Siegel S.R., Haddock B.L., Dubois A.M., Wilkin L.D. (2009). Active Video/Arcade Games (Exergaming) and Energy Expenditure in College Students. Int. J. Exerc. Sci..

[39] Verheijden Klompstra L., Jaarsma T., Strömberg A. (2014). Exergaming in older adults: A scoping review and implementation potential for patients with heart failure. Eur. J. Cardiovasc. Nurs. J. Work. Group Cardiovasc. Nurs. Eur. Soc. Cardiol..

[40] Hsu J.K., Thibodeau R., Wong S.J., Zukiwsky D., Cecile S., Walton D.M. (2011). A "Wii" bit of fun: The effects of adding Nintendo Wii® Bowling to a standard exercise regimen for residents of long-term care with upper extremity dysfunction. Physiother. Theory Pract..

[41] Shubert T.E. (2010). The use of commercial health video games to promote physical activity in older adults. Ann. Longterm Care.

[42] Theng Y.-L., Dahlan A.B., Akmal M.L., Myint T.Z. An exploratory study on senior citizens’ perceptions of the Nintendo Wii: The case of Singapore. Proceedings of the 3rd International Convention on Rehabilitation Engineering & Assistive Technology.

[43] Mazzeo R.S. Exercise and the Older Adult. ACSM CURRENT COMMENT. https://blogs.umass.edu/bodyshop/files/2009/07/exerciseandtheolderadult.pdf.

